# PAIP2 is a potential diagnostic and prognostic biomarker of breast cancer and is associated with immune infiltration

**DOI:** 10.3389/fgene.2022.1009056

**Published:** 2022-11-10

**Authors:** Chenyu Wang, Xianglai Jiang, Jiaojiao Qi, Jiachao Xu, Guangfei Yang, Chengrong Mi

**Affiliations:** ^1^ School of Clinical Medicine, Ningxia Medical University, Yinchuan, China; ^2^ Department of Ultrasound, General Hospital of Ningxia Medical University, Yinchuan, China

**Keywords:** Paip2, TCGA, biomarkers, breast cancer, immune

## Abstract

Breast cancer is the second highest incidence of cancer in the world. It is of great significance to find biomarkers to diagnose breast cancer and predict the prognosis of breast cancer patients. PAIP2 is a poly (A) -binding protein interacting protein that regulates the expression of VEGF. However, the possible role of PAIP2 in the progression of breast cancer is still unknown. RT-qRCR and Western blotting were used to verify the expression of PAIP2 in breast cancer cells and normal breast cells. The data of breast cancer samples were obtained in the TCGA database and the HPA database to analyze the expression of PAIP2 in breast cancer samples. Transwell experiment and CCK8 experiment confirmed the changes in the invasion and proliferation ability of PAIP2 after siRNA was down-regulated. Using bioinformatics technology to explore the prognostic value of PAIP2 and its possible biological function, and its effect on tumor immunity and immunotherapy. Studies have shown that PAIP2 has higher expression in breast cancer tissues and breast cancer cells. PAIP2 can promote the proliferation and invasion of breast cancer cells and has significantly high expression in higher tumor stages. The high expression of PAIP2 is associated with better OS in breast cancer patients and is negatively correlated with most chemotherapeutic drug sensitivity and IPS in cancer immunotherapy. Our study explored the potential value of PAIP2 as a biomarker for diagnosis and prognosis and may predict the efficacy of immunotherapy, providing reference for the follow-up study on the role of PAIP2 in breast cancer.

## Introduction

Cancer is an important public problem that causes death worldwide. Among them, breast cancer is one of the most common cancers, accounting for 30% of female cancers (Ferlay et al., 2021). Although early detection, early diagnosis, early treatment can reduce mortality, but once the patient is diagnosed with distant metastasis, the survival rate of the patients will be greatly reduced (Kim, 2021; Park et al., 2022). The most common site of breast cancer is the lymph node. Lymph node metastasis is also one of the important prognostic indicators of patients (Nathanson et al., 2018; To et al., 2020). According to previous studies, there are many factors affecting the mechanism of lymph node metastasis in breast cancer, such as the role of chemokines, gene expression, gastrin-releasing peptide and so on (Eccles et al., 2007; Ni et al., 2012; Hayasaka et al., 2022). Although the molecular mechanism for transfer has been identified, this process is too complex and still has the value of being studied. Therefore, it is necessary to find more factors affecting the development of breast cancer and study their expression in patients with different clinical characteristics (lymph node stage et al.).

PAIP2 (poly(A)-binding protein-interacting protein 2) mainly exists in the cytoplasm and controls mRNA stability and translation by interacting with PABP (poly(A)-binding protein). In neurons, PAIP2 is a factor affecting synaptic plasticity, memory and learning (Yoshida et al., 2006; Khoutorsky et al., 2013). In head and neck squamous cell carcinoma, PAIP2 was associated with tumor growth and apoptosis, and PAIP2 was found to control the expression of VEGF-A in head and neck cancer was controlled by PAIP2 (Onesto et al., 2006; Hauser et al., 2015). Through the mouse experiment, it can also be found that PAIP2 affects the development of germ cells (Yanagiya et al., 2010). However, the role of PAIP2 in breast cancer remains unclear. Therefore, we will study PAIP2. We use public databases and cell experiments to evaluate the role of PAIP2 expression in prognosis and prediction. And to explore the possibility of PAIP2 as a target therapy of breast cancer.

## Materials and methods

### Cell culture

 MCF-7 breast cancer cells and MCF-10a normal breast epithelial cells purchased from Procell Life Science & Technology CO., Ltd. (Wuhan, China). The MCF-7cells were cultured in DMEM (Gibco, USA) containing 10 % fetal bovine serum, and the MCF-10a cells were cultured in special medium(Procell, China). The cells were incubated at 37 °C in a 4 % CO_2_ incubator.

### Western blot

The protein components in MCF-10a and MCF-7 breast cancer cells were extracted with kit (KeyGEN BioTECH, China), and the protein concentrations were determined with BCA kit (KeyGEN BioTECH, China). Finally, the protein concentrations of different samples were normalized. In 12% SDS-PAGE gel (KeyGEN BioTECH, China) each hole sample 30 ul, working at 100 v voltage for 2 h, using PVDF membrane to transfer protein to the membrane, with 5% milk closed 90 min, washed three times after incubation of primary antibody (PAIP2, Proteintech, USA) overnight. The second antibody (β-actin, Affinity, USA) was incubated the next day and the band was exposed with ECL luminescent solution (Meilunbio, Dalian, China).

### Quantitative real-time PCR

MCF-10a and MCF-7 breast cancer cells in logarithmic growth phase were taken. The culture medium was discarded and the lysate was added. Total RNA extraction kit (Tiangen, China) was used to extract RNA. After the concentration of extracted RNA was determined, the mRNA was reversely transcribed into cDNA according to the instructions of ABScript II RT Master Mix for qPCR with gDNA Remover (ABclonal, China). At this time, the cDNA can be stored at - 80°C for a long time. The obtained cDNA was loaded according to the instructions of Genious 2X SYBR Green Fast qPCR Mix kit (ABclonal, China) and analyzed by fluorescence quantitative PCR under the operation of a Roche 480 machine. GAPDH is used as an internal reference.

### Cell proliferative assay (CCK8)

MCF-7 and MCF-7 breast cancer cells transfected with siRNA at logarithmic phase were digested with trypsin for 2 min, and then inoculated on a 96-well plate at a density of 8 × 10^4^/ml. After incubation in a 4% CO_2_ incubator at 37°C for 24 h, 48 h and 72 h, the 96-well plate was taken out, and the culture medium was changed and 10% CCK8 solution (KeyGEN BioTECH, China) was added. OD value of cells was measured after 1 h.

### Chamber migration experiment (transwell)

Cells were inoculated in an 8-μm Transwell chamber (Corning, San Diego, USA) at a density of 5×10^4^ cells per well. The cells were cultured in a 4% CO_2_ incubator at 37°C for 12 h with 750 ul 10% serum-containing medium in the lower chamber. The upper chamber liquid was discarded, fixed with 4% paraformaldehyde for 20 min, and stained with 0.1% crystal violet for 15 min. The cell migration was observed under an inverted microscope (Olympus, Japan).

### Public data collection

The expression of PAIP2 in BRCA tissues and normal breast tissues in TCGA database and GTEx database was obtained by GEPIA (Gene Expression Profiling Interactive Analysis) portal (Consortium et al., 2015; Tomczak et al., 2015; Tang et al., 2017). Immunohistochemistry (IHC) information of PAIP2 was obtained from The Human Protein Atlas (HPA) database (https://www.proteinatlas.org/) (Thul and Lindskog, 2018), and the expression of PAIP2 protein in BRCA tissue and adjacent tissues was clarified. Kaplan-Meier survival analysis was performed on breast cancer sample data using the Kaplan-Meier Plotter website (http://kmplot.com/analysis/) to clarify the correlation between PAIP2 and the prognosis of BRCA patients (Lánczky and Győrffy, 2021). The results of immunotherapy in BRCA patients were collected from The Cancer Immunome Database (TCIA), and the effect of PAIP2 on the use of immune checkpoint inhibitors was predicted by immunophenoscore (IPS) (Van Allen et al., 2015; Hugo et al., 2016).

### Analysis of gene set enrichment

Gene Ontology (GO) enrichment analysis was performed on the significant difference gene (*p* < 0.05 and |logFC| >1) between PAIP2 high expression group and PAIP2 low expression group according to the three parts of biological process (BP), cellular component (CC) and molecular function (MF) (research, 2004). The results are shown in the form of bubble diagram. At the enrichment analysis of the same time, the KEGG pathway was performed on the significantly different genes in the PAIP2 high and low expression groups, and the results are shown in the bubble diagram (Kanehisa and Goto, 2000). Secondly, the c5. go. v7.4. symbols. gmt file and the c2. cp. kegg. v7.4. symbols. gmt file were downloaded from Molecular Signatures Database (https://www.gsea-msigdb.org/gsea/msigdb/), and GO and KEGG enrichment analysis were carried out based on Gene Set Enrichment Analysis (GSEA) method (Subramanian et al., 2005; Liberzon et al., 2011; Reimand et al., 2019).

### Tumor microenvironment analysis

R software package (‘estimate’) was used to calculate the infiltration of stromal cells and immune cells in BRCA samples from TCGA database (Gentleman et al., 2004). The results were shown by StromalScore, ImmuneScore and ESTIMATEScore, and the immune cell types that were significantly correlated with PAIP2 expression level were shown (Arneth, 2019).

### Tumor mutational burden

The tumor mutational burden (TMB) in STAD patients was calculated by R software package (‘ ggplot2 ') R software package (‘ ggpubr ') and R software package (‘ ggExtra ') from TCGA database (Goodman et al., 2017; Attali and Baker, 2019). The correlation between PAIP2 expression and TMB in BRCA samples was calculated by spearman method.

### Drug sensitivity analysis

To predict the effect of PAIP2 expression on the sensitivity of tumor patients to common drug therapy, the half-maximal inhibitory concentration (IC50) of the Genomics of Drug Sensitivity in Cancer (GDSC) database (https://www. cancerrxgene.org/) was calculated using R software package (‘pRRophetic’) (Yang et al., 2012; Geeleher et al., 2014).

## Results

### Expression of PAIP2 in breast cancer

**FIGURE 1:**
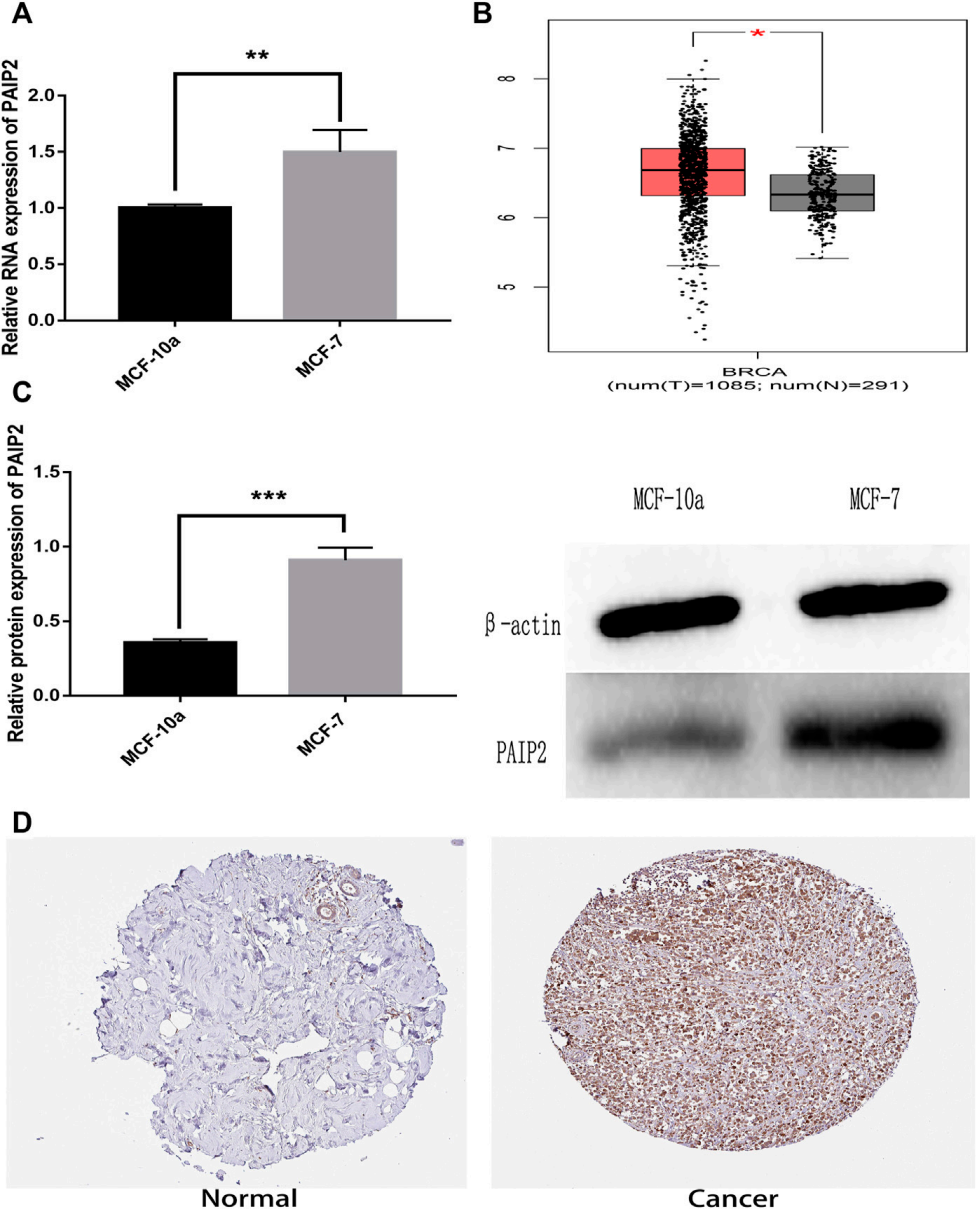
Relative expression of PAIP2 in breast cancer specimens and breast cancer cells. **(A)**: RT-qPCR showed a relatively higher expression of PAIP2 at mRNA level in breast cancer cells. **(B)**: The PAIP2 expression of BRCA tissue samples in TCGA database was higher than that of breast cancer adjacent tissues. **(C)**: Western Blot showed that the expression of the PAIP2 protein in breast cancer cells was higher than in breast cells. **(D)**: The expression level of the PAIP2 protein in breast cancer tissues was higher than in adjacent tissues from the immunohistochemical images of PAIP2 obtained from the HPA database. (* represents *p* < 0.05, * * represents *p* < 0.01 and * * * represents *p* < 0.001).

In order to verify the expression of PAIP2 in breast cancer cells *in vitro*, MCF-10a normal breast epithelial cells and MCF-7 breast cancer cells were used for comparison. The results of Western Blotting and RT-qPCR showed that the expression of PAIP2 between the 2 cells was different and had statistical significance. In breast cancer cells, the protein and mRNA expression levels of PAIP2 were higher than those in normal breast cells (Figure 1A and Figure 1C). According to the RNA-seq difference analysis of normal tissues and tumor tissues in TCGA database and GTEx database, PAIP2 is highly expressed in BRCA tissues compared with normal breast tissues (Figure 1B). The IHC results of PAIP2 in HPA database also showed that PAIP2 was higher expressed in breast cancer than in normal breast tissues at protein level (Figure 1D).

### Role of PAIP2 in proliferation and migration of breast cancer cells

**FIGURE 2:**
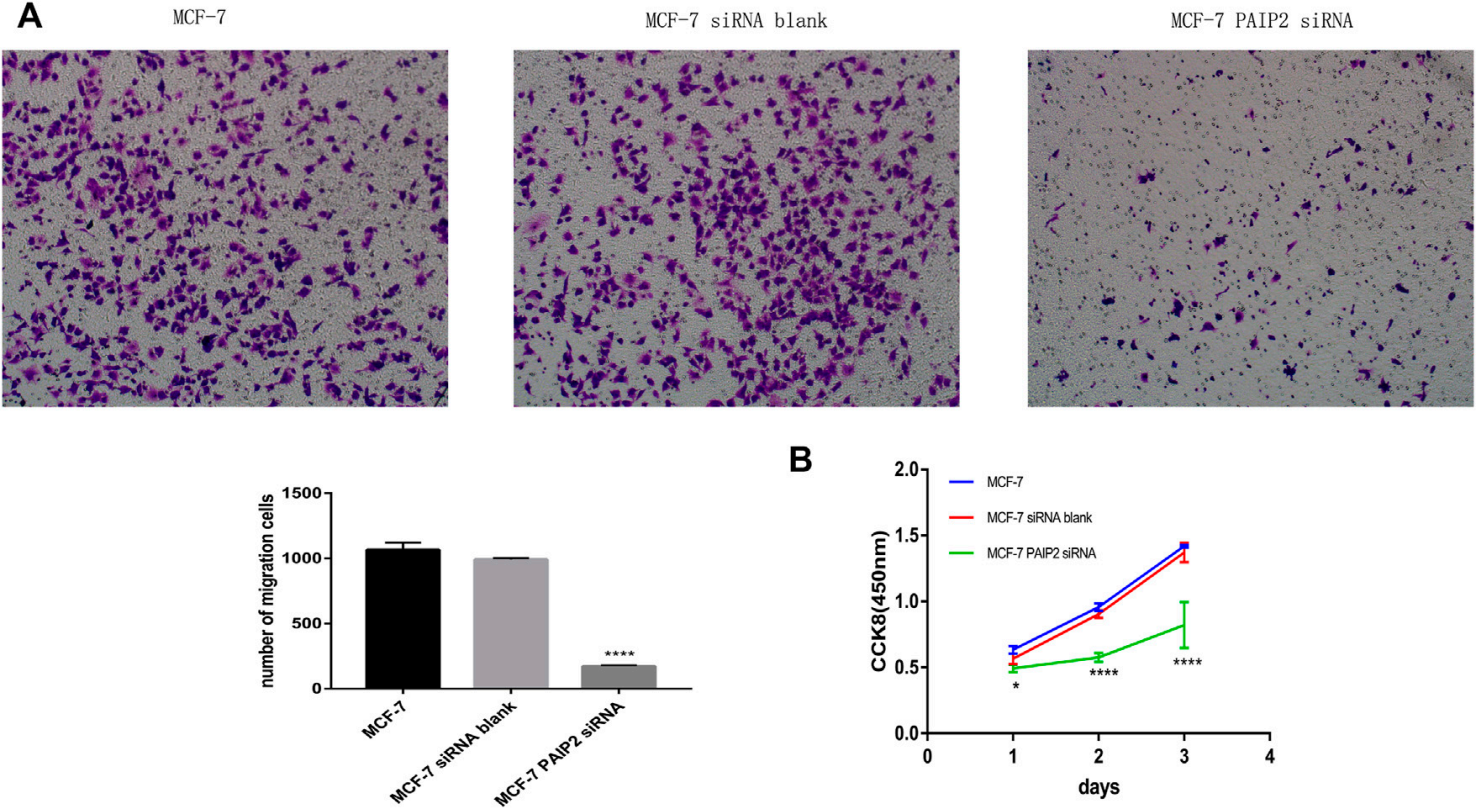
The role of PAIP2 in breast cancer cells. **(A)**: siRNA was transfected into MCF-7 breast cancer cells to reduce the expression level of PAIP2. Transwell experiment showed that the decrease of PAIP2 expression could inhibit the migration ability of cancer cells. **(B)**: The proliferation ability of MCF-7 breast cancer cells transfected with PAIP2 siRNA was lower than that of MCF-7 group and MCF-7 siRNA blank group in CCK8. The results were statistically significant (* represents *p* < 0.05, ** represents *p* < 0.01, *** represents *p* < 0.001 and **** represents *p* < 0.0001).

siRNA transfection in advance reduced PAIP2 content in MCF-7 breast cancer cells. Both proliferation and migration experiments were set to three groups of samples for comparison, namely, MCF-7 group, MCF-7 siRNA blank group and MCF-7 siRNA PAIP2 group. CCK8 results showed that the decrease of PAIP2 expression would help to reduce the proliferation of cancer cells, and it was statistically significant (Figure 2B). The proliferation effect of blank group and MCF-7 group was similar. PAIP2 was also helpful for cancer cell migration in Transwell chamber migration experiment, which was also statistically significant (Figure 2A).

### Correlation between PAIP2 expression and clinical features

**FIGURE 3:**
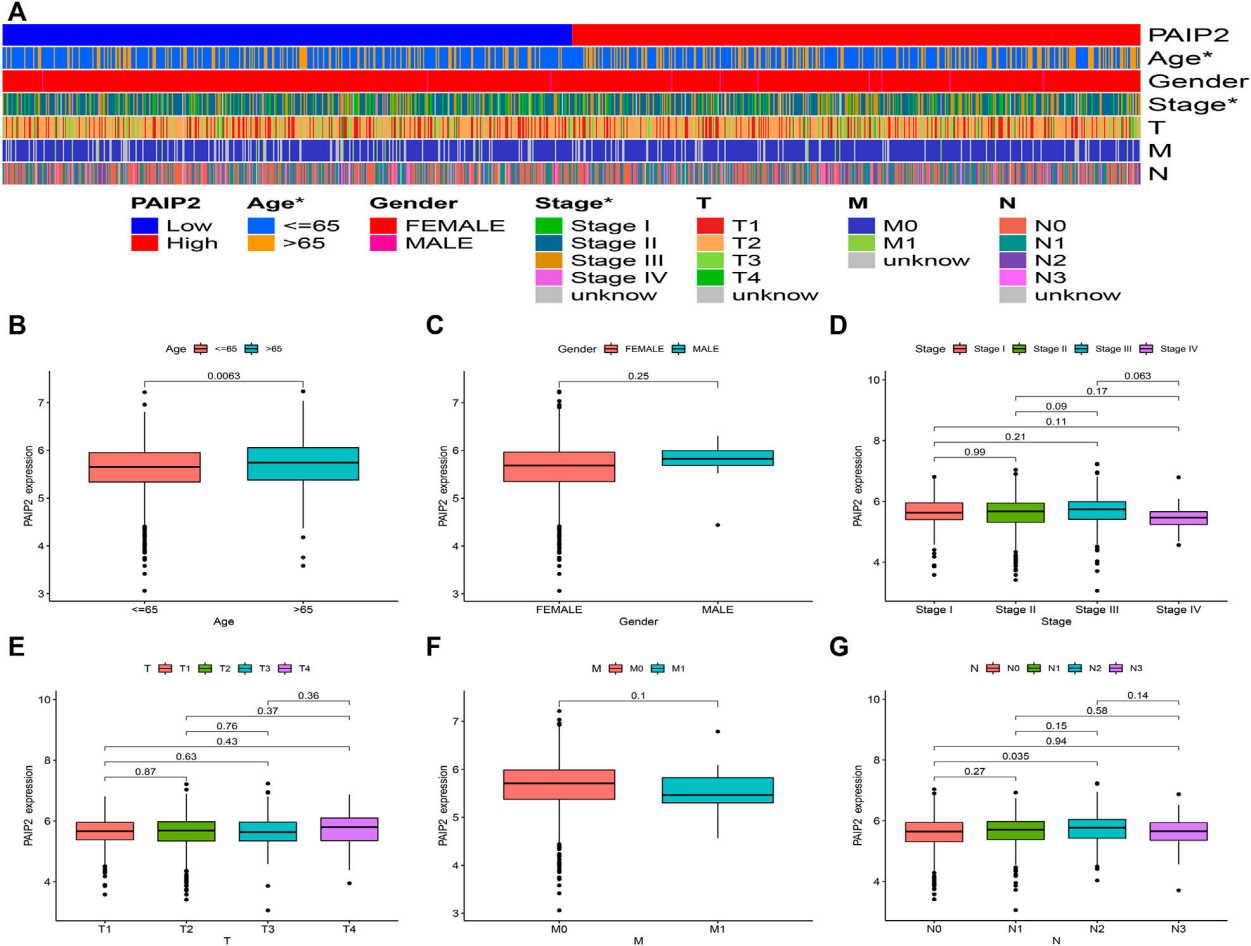
Correlation between PAIP2 expression and clinical features. **(A)**: The expression of PAIP2 was significantly correlated with age and stage. PAIP2 expression in different age **(B)**, gender **(C)**, stage **(D)**, stage T **(E)**, stage M stage **(F)** and N stage **(G)**.

In order to understand the relationship between PAIP2 and different clinical parameters of BRCA patients, we statistically analyzed the mRNA expression of PAIP2 in BRCA patients with different clinical characteristics (Figures 3A-G). PAIP2 expression was significantly correlated with age and stage in BRCA patients (Figure 3A) and was significantly higher in elderly patients (>65 years) (Figure 3B) and N2 patients (Figure 3E).

### The prognostic value of PAIP2

**FIGURE 4:**
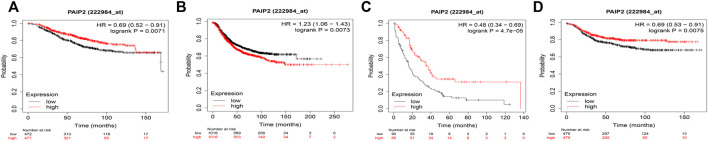
Correlation between PAIP2 and prognosis of breast cancer patients. **(A)**: The high expression of PAIP2 is associated with better OS in breast cancer patients. **(B)**: High expression of PAIP2 is associated with poor RFS in patients with breast cancer. **(C)**: The high expression of PAIP2 was associated with better PPS in breast cancer patients. **(D)**: The high expression of PAIP2 is associated with better DMFS in breast cancer patients.

Kaplan-Meier survival analysis showed that high PAIP2 expression was significantly correlated with better OS (HR = 0.36, 95% CI: 0.52–0.91, *p* = 0.0071) (Figure 4A), high PAIP2 expression was significantly correlated with poor RFS (HR = 1.23, 95% CI: 1.06–1.43, *p* = 0.0073) (Figure 4B), high PAIP2 expression was significantly correlated with better PPS (HR = 0.48, 95% CI: 0.34–0.69, *p* = 4.7e - 05) (Figure 4C) and high PAIP2 expression was significantly correlated with better DMFS (HR = 0.36, 95% CI: 0.53–0.91, *p* = 0.0075) (Figure 4D).

### PAIP2-related enrichment analysis

**FIGURE 5:**
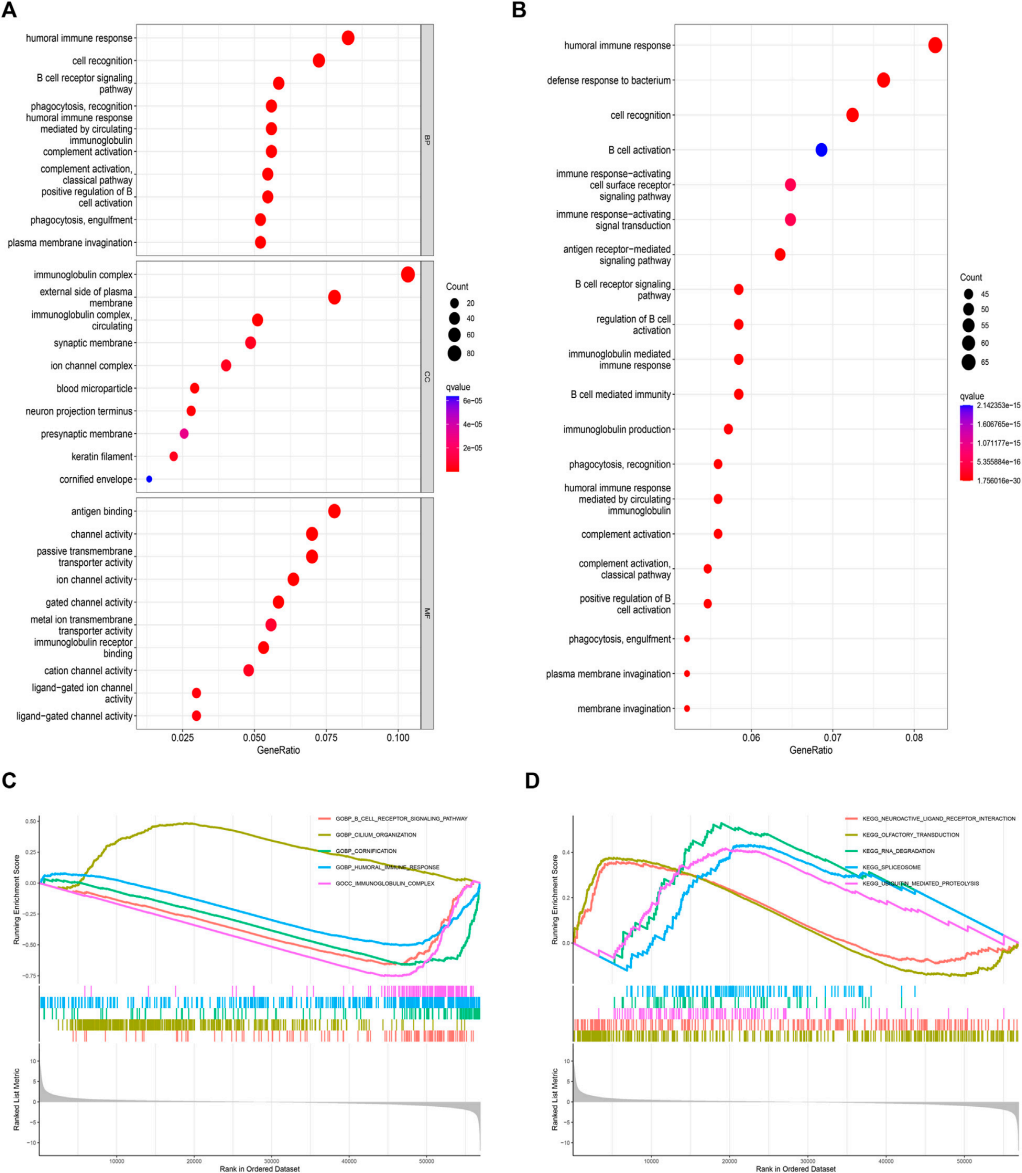
Enrichment analysis of two groups of differentially expressed genes in PAIP2. **(A)**: GO enrichment analysis of differential genes. **(B)**: KEGG enrichment analysis of differential genes. **(C)**: GSEA analysis based on GO analysis. **(D)**: GSEA analysis based on KEGG analysis. (Circle size represents the proportion of genes in this biological process or signal pathway).

GO enrichment analysis obtained the top 10 BP, CC and MF that PAIP2 may participate in, and found that PAIP2 mainly participated in the humoral immune response, cell recognition and b cell receptor signaling pathway in BP. Mainly participated in immune globulin complex, external side of plasma membrane and immune globulin complex, circulating in CC, and Mainly participated in antigen binding, channel activity and passive transmembrane transporter activity in MF (Figure 5A). Through KEGG enrichment analysis, it was found that PAIP2 was mainly involved in the humoral immune response, defense response to bacteria, cell recognition, B cell activation, Immune response-activating cell surface receptor signaling pathway and immune response-activating signal transmission (Figure 5B). The results of GSEA showed that the biological process of B cell receptor signaling pathway was active in the PAIP2 high expression group, and the biological processes of cilium, cornification, immune response and immunoglobulin complex were active in the PAIP2 low expression group (Figure 5C). It was also clear that neuroactive ligand receptor interaction, olfactory transduction, RNA degradation, spliceosome and ubiquitin mediated proteolysis were active in PAIP2 high expression group (Figure 5D).

### Correlation between PAIP2 and tumor microenvironment

**FIGURE 6:**
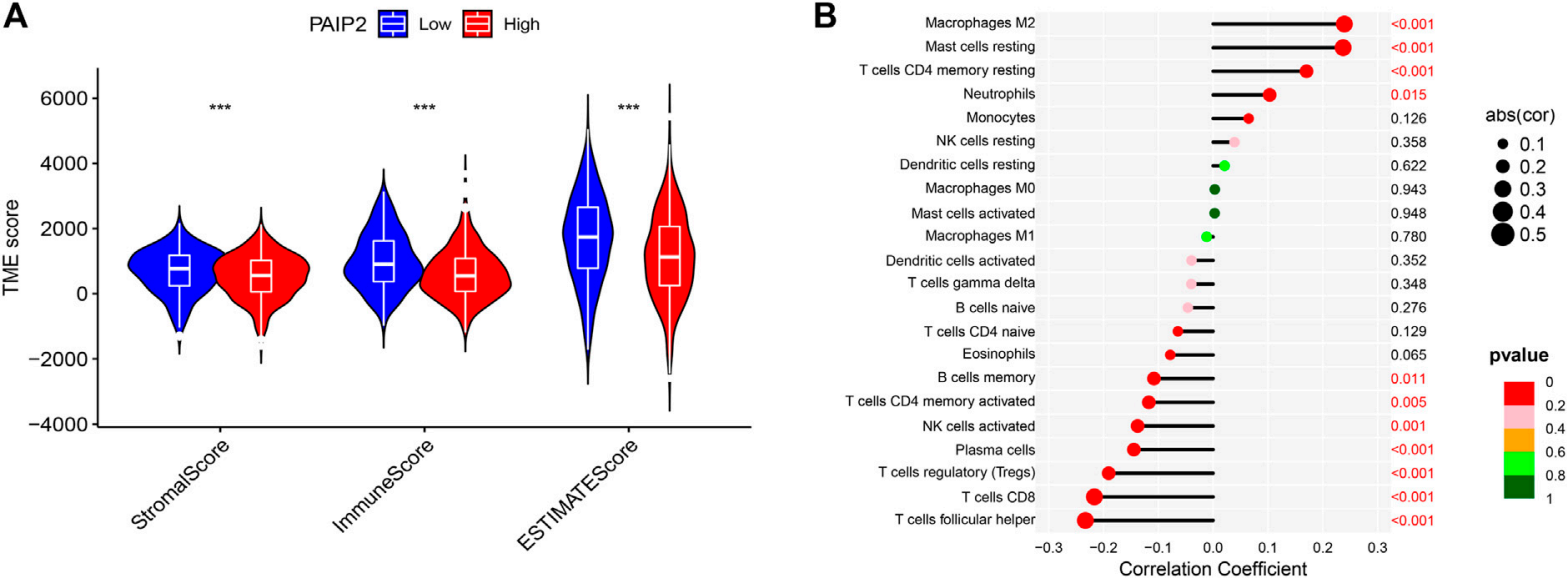
Tumor microenvironment score and immune cell infiltration. **(A)**: StromalScore, ImmuneScore and ESTIMATEScore of PAIP2 low expression group were significantly higher than those of PAIP2 high expression group (*represented *p* < 0.05, ** represented *p* < 0.01 and *** represented *p* < 0.001). **(B)**: The expression of PAIP2 was significantly positively correlated with the infiltration of Macrophages M2, Mast cells resting, T cells CD4 memory resting and Neutrophils. It was significantly negatively correlated with T cells follicular helper, T cells CD8, T cells regulatory, plasma cells, NK cells activated, T cells CD4 memory activated and B cells memory.

According to the results of the analysis of the estimation algorithm, StromalScore, ImmuneScore and ESTIMATEScore of PAIP2 high expression group were significantly lower than those of the PAIP2 low expression group (Figure 6A). The expression of PAIP2 was significantly positively correlated with the infiltration of Macrophages M2, Mast cells resting, T cells CD4 memory resting and Neutrophils. It was significantly negatively correlated with T cells follicular helper, T cells CD8, T cells regulatory, plasma cells, NK cells activated, T cells CD4 memory activated and B cells memory (Figure 6B).

### Correlation between PAIP2 and tumor immunotherapy

**FIGURE 7:**
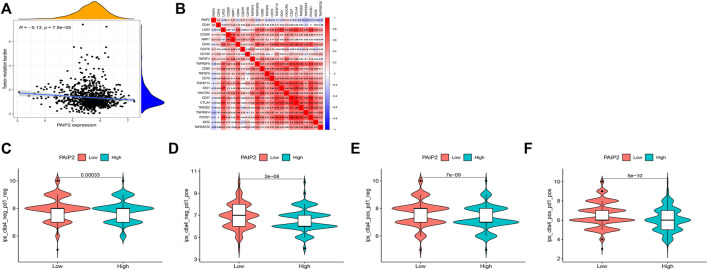
Correlation between PAIP2 and tumor immunity. **(A)**: The expression of PAIP2 was significantly negatively correlated with TMB in breast cancer. **(B)**: PAIP2 was significantly associated with 22 immune checkpoint genes in breast cancer (bule represents a negative correlation, red represents a negative correlation). **(C–F)**: The IPS of low PAIP2 expression group was higher than that of high PAIP2 expression group.

TMB is an important predictor of tumor immunotherapy. The correlation analysis between PAIP2 expression and TMB shows that PAIP2 expression is negatively correlated with TMB in breast cancer (Figure 7A). From the correlation analysis between PAIP2 and the gene expression levels of immune checkpoints, it can be found that PAIP2 was significantly positively correlated with the gene expression levels of eight immune checkpoints, and significantly negatively correlated with the gene expression levels of 14 immune checkpoints (Figure 7B). The IPS data of BRCA patients from TCIA database showed that PAIP2 low expression group had higher IPS than PAIP2 high expression group, and had better response to immune checkpoint inhibitors (Figures 7C–F).

### The correlation between PAIP2 and chemotherapeutic drug sensitivity

**FIGURE 8:**
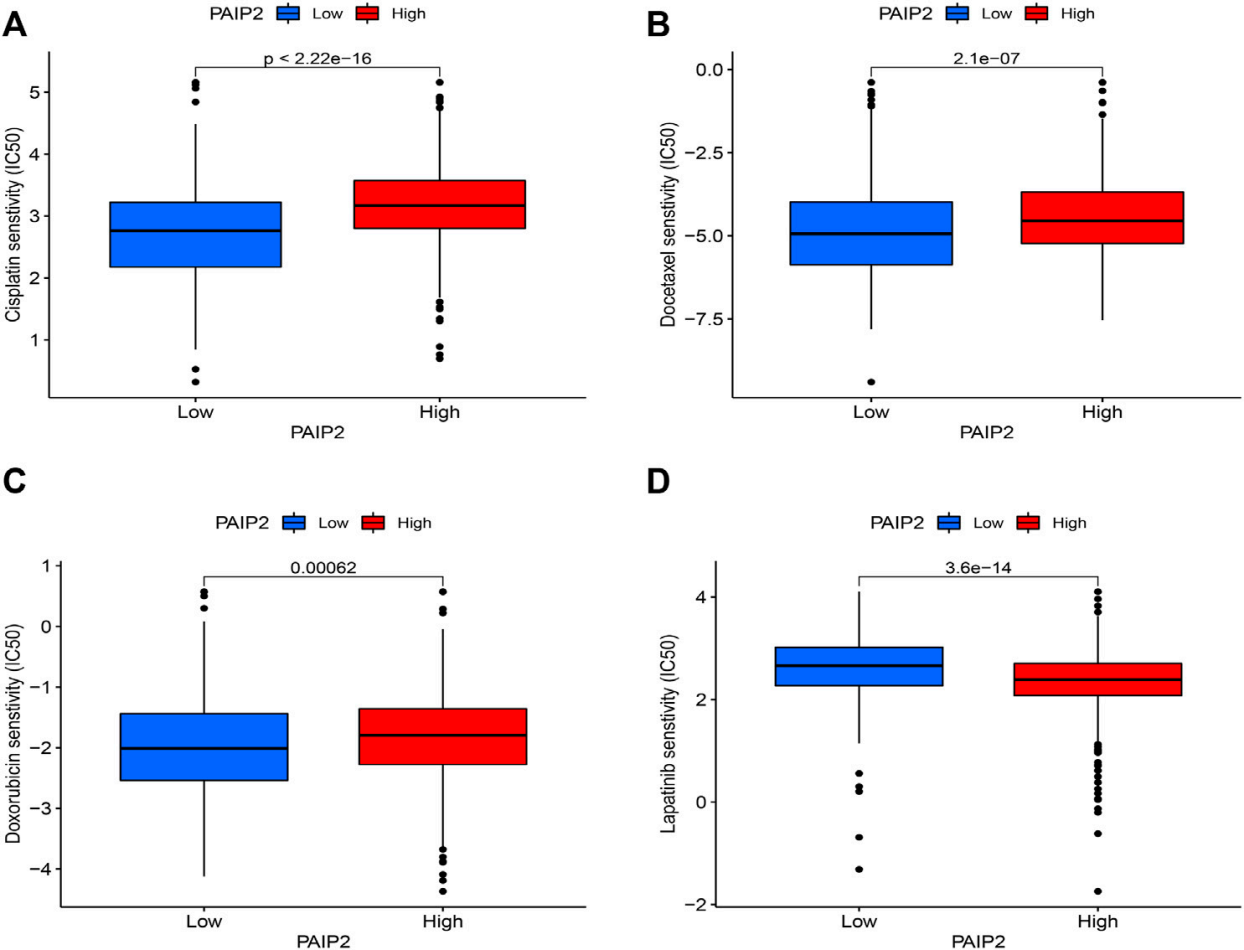
Correlation between PAIP2 and drug sensitivity. **(A)**: The expression of PAIP2 was positively correlated with the IC50 of Cisplatin and negatively correlated with the sensitivity of Cisplatin. **(B)**: PAIP2 expression was positively correlated with the IC50 of Docetaxel and negatively correlated with the sensitivity of Docetaxel. **(C)**: PAIP2 expression was positively correlated with Doxorubicin IC50 and negatively correlated with Doxorubicin sensitivity. **(D)**: PAIP2 expression was negatively correlated with IC50 of Lapatinib and positively correlated with sensitivity of Lapatinib.

Based on the pRRophetic algorithm and the limma algorithm, the IC50 of the PAIP2 high expression group and the low expression group in the GDSC database was compared. The results showed that the IC50 of 119 drugs was significantly correlated with the expression level of PAIP2. The PAIP2 table was significantly positively correlated with the IC50 of 82 chemotherapeutic drugs such as Doxorubicin (Figure 8C), Docetaxel (Figure 8B), and Cisplatin (Figure 8A), and significantly negatively correlated with 37 chemotherapeutic drugs such as Lapatinib (Figure 8D). The details are shown in Annex.1.

## Discussion

By analyzing the data from the public database, RT-qPCR and Western blotting, we clarified that PAIP2 was highly expressed in breast cancer cells and breast cancer tissues at mRNA and protein levels, compared with normal breast cells and adjacent tissues, which was consistent with the high expression detected by previous researchers in human head and neck cancer (Onesto et al., 2006), suggesting that PAIP2 had potential value as a diagnostic biomarker for breast cancer. According to the results of prognostic analysis, PAIP2 was a risk factor for RFS in breast cancer patients (HR = 1.23, 95% CI: 1.06-1.43). The correlation analysis of clinical characteristics showed that PAIP2 expression was higher in stage N2 than in N0 stage. Transwell and CCK8 experiments also confirmed that PAIP2 could promote the invasion and proliferation of breast cancer cells, which suggested that PAIP2 was a risk factor for promoting the progression of breast cancer. PAIP2 is involved in a variety of biological processes such as cell proliferation and differentiation (Kachaev et al., 2018), and can be used as an effector of cell growth in *Drosophila* (Roy et al., 2004). PAIP2 is also a VEGF mRNA 3 ' -UTR interacting protein, can regulate the expression of VEGF, such as in head and neck cancer (Onesto et al., 2004; Onesto et al., 2006). Therefore, we speculate that PAIP2 may promote breast cancer by affecting VEGF, a signaling pathway that is clearly shown to play an important role in breast cancer growth and metastasis of breast cancer. Interestingly, although it is clear that PAIP2 can promote breast cancer, from a longer-term perspective of OS and PPS, the PAIP2 high expression group is significantly associated with longer OS, PPS, and DMFS in breast cancer patients. Even if more clinical trials are needed to support PAIP2 expression as a protective factor for OS, PPS and DMFS in STAD patients, it also shows the potential value of PAIP2 as a prognostic biomarker for breast cancer patients. In the further GSEA, we found that the main active pathway in the PAIP2 low expression group was the immune response and B cell receptor signaling pathway. In the study, we also found that the Stromal Score, Immune Score and ESTIMATEScore in the PAIP2 low expression group were significantly higher than those in the PAIP2 high expression group. PAIP2 was positively correlated with the infiltration of four immune cells and negatively correlated with the infiltration of seven immune cells. Immune infiltrations in TME have been proved to be one of the key factors in the development of tumors, and it is also the factor that influences cancer treatment (Harbeck et al., 2019). The mechanism of TME in tumors has not been clarified, and the therapeutic effect of immunotherapy on breast cancer has not been determined (Zhang and Zhang, 2020). However, by observing these indicators of TMB and IPS in breast cancer immunotherapy, we can also find that the expression of PAIP2 is negatively correlated with TMB and IPS in cancer immunotherapy, suggesting that PAIP2 has potential value in predicting the therapeutic effect of breast cancer immunotherapy (Charoentong et al., 2017; Samstein et al., 2019). We found that high expression of PAIP2 was associated with possibly worse immunotherapy, and it was also found to be negatively correlated with the sensitivity of 82 chemotherapeutic drugs such as Doxorubicin, Docetaxel, and Cisplatin. In general, although there is no specific mechanism study, our study has clarified the high expression of PAIP2 in breast cancer cells and tissues, and has the role of promoting the proliferation and metastasis of breast cancer cells. In bioinformatics analysis, PAIP2 is found to be an influencing factor for the prognosis of breast cancer patients and may also have potential value in predicting the effect of immunotherapy for breast cancer.

## Conclusion

We found that PAIP2 has potential value as a diagnostic biomarker and prognostic marker for breast cancer and may predict the effect of immunotherapy for breast cancer.

Annex 1: Correlation between PAIP2 expression and drug sensitivity. A total of 119 chemotherapy IC50 were significantly correlated with the expression of PAIP2, of which 82 chemotherapy IC50 were significantly positively correlated with the expression of PAIP2, and 37 chemotherapy IC50 were significantly negatively correlated with the expression of PAIP2.

## Data Availability

The datasets presented in this study can be found in online repositories. The names of the repository/repositories and accession number(s) can be found in the article/Supplementary Material.
