# Supplementary figures and images for: PAIP2 is a potential diagnostic and prognostic biomarker of breast cancer and is associated with immune infiltration
Source: Front Genet. 2022 Nov 10;13:1009056. doi: 10.3389/fgene.2022.1009056 (PMC9685164; doi:10.3389/fgene.2022.1009056)

MCF-10a MCF-7 siRNA MCF-7

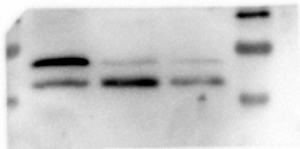

Supplement: Supplementary file 1 [file DataSheet2.PDF]

MCF-10a MCF-7 siRNA MCF-7

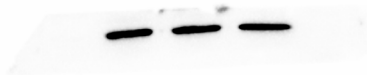

Supplement: Supplementary file 3 [file DataSheet1.PDF]
